# Single Red Blood Cell Hydrodynamic Traps via the Generative Design

**DOI:** 10.3390/mi13030367

**Published:** 2022-02-26

**Authors:** Georgii V. Grigorev, Nikolay O. Nikitin, Alexander Hvatov, Anna V. Kalyuzhnaya, Alexander V. Lebedev, Xiaohao Wang, Xiang Qian, Georgii V. Maksimov, Liwei Lin

**Affiliations:** 1 Data Science and Information Technology Research Center, Tsinghua Berkeley Shenzhen Institute, Tsinghua University, Shenzhen 518055, China; georgii@berkeley.edu; 2 Mechanical Department, University of California in Berkeley, Berkeley, CA 94703, USA; lwlin@berkeley.edu; 3 NSS Lab, ITMO University, 197101 Saint-Petersburg, Russia; nnikitin@itmo.ru (N.O.N.); alex_hvatov@itmo.ru (A.H.); anna.kalyuzhnaya@itmo.ru (A.V.K.); 4 Machine Building Department, Bauman Moscow State Technical University, 105005 Moscow, Russia; mr.aleksanderl@yandex.ru; 5 Shenzhen International Graduate School, Tsinghua University, Shenzhen 518055, China; 6 Biophysics Lab, Biology Department, Moscow State University, 119192 Moscow, Russia; gmaksimov@mail.ru; 7 Physical metallurgy Department, Federal State Autonomous Educational Institution of Higher Education, National Research Technological University, MISiS, 119049 Moscow, Russia

**Keywords:** microfluidics, cell trap, RBC, evolutionary algorithm, generative design, artificial intelligence

## Abstract

This paper describes a generative design methodology for a micro hydrodynamic single-RBC (red blood cell) trap for applications in microfluidics-based single-cell analysis. One key challenge in single-cell microfluidic traps is to achieve desired through-slit flowrates to trap cells under implicit constraints. In this work, the cell-trapping design with validation from experimental data has been developed by the generative design methodology with an evolutionary algorithm. L-shaped trapping slits have been generated iteratively for the optimal geometries to trap living-cells suspended in flow channels. Without using the generative design, the slits have low flow velocities incapable of trapping single cells. After a search with 30,000 solutions, the optimized geometry was found to increase the through-slit velocities by 49%. Fabricated and experimentally tested prototypes have achieved 4 out of 4 trapping efficiency of RBCs. This evolutionary algorithm and trapping design can be applied to cells of various sizes.

## 1. Introduction

Microfluidic devices are indispensable for studying behaviors of single living cells, such as cytological, mechanical, and electrical responses for potential applications ranging from early disease diagnosis to drug testing. To study single-cell activities, microfluidics trapping systems are important platforms; several approaches have been reported previously, such as acoustic [1,2], dielectrophoretic [3,4], hydrodynamic [5,6,7,8], magnetic [9,10], and optical trapping schemes [11,12]. Hydrodynamic trapping of single cells happens within a microfluidic channel, where the channels’ geometries and flowrates require careful study and optimization. Hydrodynamic trapping utilizes mechanical barriers or arrays for the separation of target particles from the main flow. Separated particles are retained within the hydrodynamic traps for further analyses by employing various principles, such as vortices-based trapping (centrifugation assisted, cavitation microstreaming, hydrodynamic tweezers), cross streamed (viscoelastic focusing, inertial migration, dean flow and deformability selective cell separation), and external controlled approaches (pneumatic valves, PID controllers, eddy currents, electro-magnetic fields, acoustics) [6]. The advantages of hydrodynamic processing are the ease to implement the inertial focusing of enhanced cell separation and sorting with narrowed sheathed flows. As a disadvantage, the hydrodynamic single cell platform may produce stress on cell samples, and is reported to alter molecular mechanisms, and inhomogeneity issues [13,14,15,16]. The geometric design of hydrodynamic single cell trapping belongs to the category of “wet fluid-structure interaction (FSI)”; a standard approach is the topology optimization through gradient-based methods [17,18].

In this work, we approached the FSI design problem by using the evolutionary algorithm. It allowed us to extend the ‘classical’ statement of the topology optimization and explore more comprehensive designs with extended variability [19]. In general, the topology generative design has no a priori assumptions on the form of the initial design, i.e., optimization starts ‘from scratch’. The idea of evolutionary generative design allows the algorithm to extend the possible design from the creation and improvement of the digital twins of real-world objects [20]. Researchers have also successfully applied evolutionary algorithms and other AI methods in different areas, including studies for coastal structures [21], mathematical models [22], architecture [23], and drug designs [24]. However, the adaptation of this approach to the hydrodynamic cell trappings has specific factors that should be taken into consideration. As an example, we have developed task-specific evolutionary operators, validation rules, objective functions, and post-processing procedures in this work. In addition, the parallelization of computations has been implemented due to the high computational cost of the hydrodynamic simulations. Here, the micro hydrodynamic traps were designed with a unique feature to trap RBCs (red blood cells) in channels while keeping it suspended to allow fluidic flows. The trapping chamber was designed with a specific implementation evolutionary algorithm. The prototype devices have been fabricated and tested using frog RBCs as the living cells for validations.

## 2. Materials and Methods

### 2.1. Single Cell Traps

**Figure 1:**
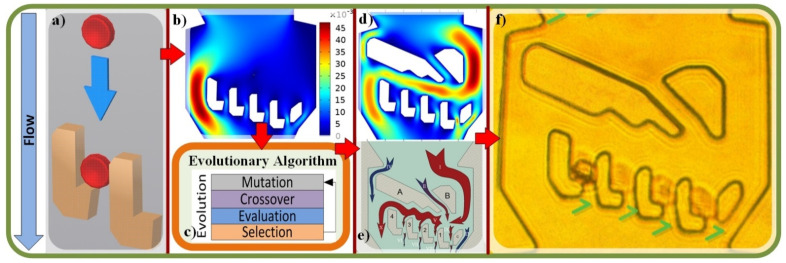
The overall device design sequence. Blue arrow indicates the flow direction; (**a**) the single RBC trapping principle; (**b**) an example of the chamber with trapping structure deign with zero trapping chance due to the low through-trap flow rate; color bar corresponds to the flow velocity distribution (**c**) the evolutionary algorithm which generated necessary flow obstacles to obtain high RBC trapping chances; (**d**) COMSOL simulation of the velocity gradient in the final geometry of the trapping chamber (inflow velocity = 0.01 m/s, no slip boundary condition); (**e**) flow schematics in the trapping chamber; (**f**) experimental results of the prototype system following the evolutionary algorithm design to trap RBCs; green “V”s mark the successfully trapped cells in each slot.

The goal of this work is to create single-cell traps by an evolutionary-based generative design. Figure 1a shows the design principle for the single RBC trapping scheme and Figure 1b is an example for the chamber design with the trapping structure of the zero trapping chance due to the low through-trap flow. Figure 1c demonstrates the result from the evolutionary algorithm, which generated the necessary flow obstacles to obtain the high trapping probability. The COMSOL simulation results are displayed in Figure 1d, showing the velocity gradient in the final design with inflow velocity of 0.01 m/s and the no slip boundary condition. Figure 1e shows the flows schematics in the trapping chamber and Figure 1f presents the experimental results of the prototype system following the evolutionary algorithm design to trap RBCs.

The properties and constraints of the microfluidic single-RBC traps are targeted to have 4 erythrocytes trapped within one FOV (field of vision) while keeping the flow streams around RBCs. This is different to erythrocytes trapped in cavity, pocket, and well-like structures with little or no fluid flows [8,25,26]. The basic assumptions include: a single phase Navier–Stokes flow, steady-state, and no-slip boundary condition. The rheological parameters used in this work include: the kinematic viscosity of 3.3 mm^2^/s; dynamic viscosity of 0.0035 kg/ms; and the fluid density of 1060 kg/m^3^ (blood density). PDMS (polydimethylsiloxane) was chosen as the material with built-in properties in the COMSOL Multiphysics library. For all the simulations in COMSOL Multiphysics, the following built-in meshing parameters were used. We set sequence type as physics-controlled mesh and element size as finer, respectively.

Frog RBCs were chosen as the trapping object based on their good availability and bigger nucleated RBCs were chosen for good trapping visualizations. To implicitly account for the number of cells trapped, three physical parameters were calculated for each design. The *CRV* and *CRL* parameters were used as the constraints to avoid the cases where the cells are destroyed by the flow. The *CRV* (curvature of rotated vector fields) reflects the flow curvature level. It takes the cross-section of a channel flow and cuts it into multiple pieces with probed values for each piece, and then performs a summation of all velocities from all the pieces of the cross-section as:

(1) $$CRV=\underset{\Omega }{\iint }abs\left[\frac{u^{2}*\frac{\partial v}{\partial x}-v^{2}\frac{\partial u}{\partial y}+uv*\left(\frac{\partial v}{\partial y}-\frac{\partial u}{\partial x}\right)}{{\left(u^{2}+v^{2}\right)}^{3/2}}\right]dxdy$$

where *u* is the horizontal velocity and *v* is the vertical velocity. Second, the *CRL* reflects rapid changes in the flow channel and calculates the maximum of velocity and velocity gradient in the designated area. It takes the sum of the maximum values of the y derivative of *u* and *x* derivative of *v* as:

(2) $$CRL=\underset{\Omega }{\max}\left(\frac{\partial u}{\partial y}\right)+\underset{\Omega }{\max}\left(\frac{\partial v}{\partial x}\right)$$

Third, *TVR* is used as an objective function and maximized during the optimization process. *TVR* is the flow ratio in trapping slits and those of non-trapping channels to ensure the cells can be trapped as the objective of topology optimization:

(3) $$TVR=\frac{\sum_{k=1}^{4}v_{k}}{v_{PD}+v_{main}}\;$$

where $v_{k}$ is flow velocity (m/s) in the trapping slit, k=1, 2, 3, 4; $v_{PD}$ is the flow velocity in the pressure dropping channel, and $v_{main}$ is the flow velocity in the main output channel.

**Figure 2:**
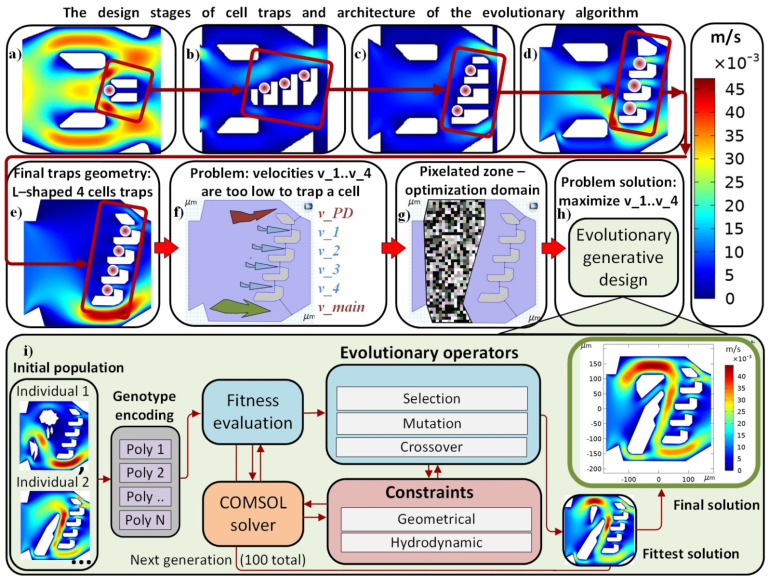
The design stages of cell traps and the architecture of the evolutionary algorithm. Flow direction from left to right. Red circles symbolize the RBCs. (**a**–**e**) are stages of cell trapping structures: each L-shape trap traps one cell; red rectangle highlights the design changes of the trapping slits; (**f**)—problem statement: velocities v_1…v_4 through slits have near-zero values, and almost zero flux pass through these slits; thus it is incapable of luring a cell by this configuration. Topology optimization is needed (TVR, Equation (2)) to increase the flow velocity in the slits to insure cell trappings; Red, blue, and green colors indicate the flow (**g**)—topology optimization domain (marked pixilated zones); (**h**,**i**)—architecture of the evolutionary algorithm with the final design of the trapping chamber.

Designs from Figure 2a to Figure 2b has improved efficiency by increasing the number of trapping slits to 3 with the new L-shape structure where the opening width of the slit is close to the width of the cells. Furthermore, the slits were placed at 90-degrees against the flow direction with an increased height, as shown to allow cell trappings. The flow simulation results show poor flow gradient to trap cells. Figure 2c shows the L-shape traps placed in the same direction of the flow to trap cells, while the flow velocities in the slits are nearly zero, due to the high flow resistance. To further improve the cell trapping efficiency, a more advanced version of L-shaped traps with geometric alternations were designed to increase the flow velocity in the slits, as shown in Figure 2d. In the later experimental sections, the typical length and breadth of the nucleated erythrocytes of frog were 19.8 ± 1.5 μm and 8.6 ± 0.3 μm, respectively. As such, the slit width was chosen as 11 μm. Qualitatively, the L-shaped traps possessed the built-in 90-degree microfluidic obstacle to create weak flows for cells to flow into the trap and remain there. The stagnation-point flows and Moffatt-type vortices have been studied in the trapping slits as part of the design considerations. 

Figure 2e shows the final design for the L-trap with 4 traps within 1 FOV. The top of the chamber has a 21 μm-wide pressure dropping channel (red arrow in Figure 2f), which helps to slow down the flow along the traps, while an erythrocyte can pass through easily. The design also removes the two upstream rectangular trapezoidal shape structures and becomes the initial design for the topology optimization process. Figure 2f shows the specific flow velocity in the trapping slits used in Equation (3). The pixelated zone in Figure 2g is the optimization domain where the evolutionary algorithm was used by placing polygons to optimize flow velocities under the constraints of Equations (1)–(3). 

Figure 2h,i are the architecture of the evolutionary algorithm with major components and stages, including initial population examples, evolutionary loops and the final solution which satisfies the objective function and constraints. The optimization pipeline presented in Figure 2i is organized accordingly. First, the initial population was set in a random way. Each design in this population was represented as a list of polygons; the values of fitness for the designs were evaluated using the COMSOL simulator. The evolutionary operators were used to modify the designs. Next, modified designs were used in the selection stage; the solution obtained after the last iteration represented the best design.

### 2.2. Evolutionary Algorithm

The initial trapping slit designs from the previous section have flow velocities of less than 0.009 m/s (simulated with COMSOL) with zero trapping probability. The generative design creates obstacles in the chamber to increase the flow velocities in slits. Our design approach refers to the alternative scheme of the topology optimization—the intelligent field of generative design [22], by using artificial intelligence (AI) and machine learning (ML) methods to create a diversity of variants that have adequate values of the objective function, while satisfying all limitations. 

The evolutionary optimization of the cell trapping topology has been implemented using the concept of genetic algorithms with continuous numerical genotype encoding [27]. The pipeline of the algorithm’s implementation is presented in Figure 2. As can be seen, the evaluation of the fitness function was based on the COMSOL model. The selection stage was based on the binary tournament algorithm. The pseudocode of the evolutionary algorithm is presented in Algorithm 1. It consists in a procedure for generating initial populations, evolutionary operators, constraint validations, and connectors to the hydrodynamic model. The software implementation of the algorithm has been done in Python 3.8 and is available as a part of the GEFEST framework (https://github.com/ITMO-NSS-team/GEFEST; accessed on 23 December 2021).
***Algorithm 1:** Evolutionary algorithm for cell trap design***input: *params* = set of hyperparameters for evolutionary algorithm (population size, number of populations, etc)** ***constraints* = set of constraints for cell trap** **output: Best found cell trap design** ***▸Generate random initial population*** ***pop*←InitPopulation(*params.pop_size, constraints*)** **while not IsFinished(*params.num_pop*) do** ***offsprings*←Reproduce(*pop*, *constraints*)** ***pop.fitness*←Fitness(*pop*, *constraints*)** ***pop*←TournamentSelection(*offsprings*)** **return Best(*pop*)** **procedure Reproduce:** **input: *pop*, *constraints*** **output: *offsprings*** **while not Validate(*constraints*)** ***modify cell traps designs*** ***offsprings*←Crossover (*pop*)** ***offsprings*←Mutation (*pop*)** ***return offsprings*** **procedure Fitness:** **input: *pop*, *constraints*** **output: fitness values for each individual** ***fitnesses={}*** **for *ind* in *pop*:** **if Validate(*individual*, *constraints*)** ***run sim for cell trap described in genotype*** ***fitnesses[ind]*←COMSOL_Sim(*ind*)** **else** ***fitnesses[ind]*←0** ***return fitnesses***

**Figure 3:**
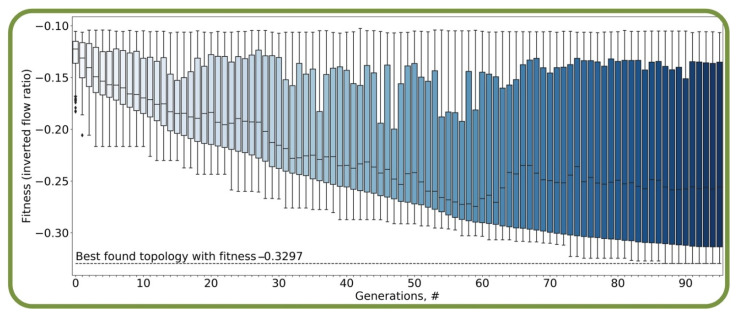
The convergence of the fitness function values (the function that is used to estimate how close a given design solution is to the specified aim) during the evolutionary optimization of cell traps for the 100 generations (#, iterations of evolution). The boxplots represent the diversity of the solutions in each population: the centerline of each box represents the median of the fitness distribution in each population, the boundaries of the box—25 and 75 percentiles of the same distribution, and the additional lines, represent the minimum and maximal values of fitness. The solution with the best fitness in all populations is highlighted with the dashed line. It can be seen that the quality of solutions improved steadily during the optimization. The shade of the boxplots’ color depends on the number of a generation.

The convergence of the evolutionary search for the best topology is presented in Figure 3, where the convergence of the fitness function was calculated by comparing a given design solution to the specified aim for the 100 generations (iterations of evolution). We show that the diversity was successfully preserved even in the late generations of the algorithm. In addition, the optimization was not converging to local minima and the final solution was constantly improving without long stagnation.

The genotype of the designs was represented in the vector form. It consisted of N polygons with $K_{i}\in \left[3,\ldots ,N_{vert}\right]$ vertices, where *i* = 1, …, *N*. The objective function was based on inverted values of the flow ratio. Several custom operators for the mutation, crossover, and initialization of the initial population were implemented and the competition selection was used to preserve diversity. There are different mutations: rotation, rescaling or movement of entire geometric polygons, or single nodes in it. The crossover was implemented at the polygon level to allow for the combination of promising solutions to obtain a more effective one.

### 2.3. Fabrication

PDMS microfluidics chips were fabricated in a clean room by a typical soft lithography method and were glued to a glass substrate. The photolithography and molding were made with a negative photoresist SU-8 2025 (25 μm height). We used a Sylgard 186 Silicone Elastomer Kit for the PDMS slab fabrication.

### 2.4. Bio-Samples Preparation

Samples of frog blood were purchased from YuanXieShengWu, Shanghai, PRC (LOT:H13J9Q65425,) in accordance with the Tsinghua University (Graduate School in Shenzhen) Ethics Committee.

## 3. Results and Discussion

### 3.1. Optimal Designed Micfluidic Traps

**Figure 4:**
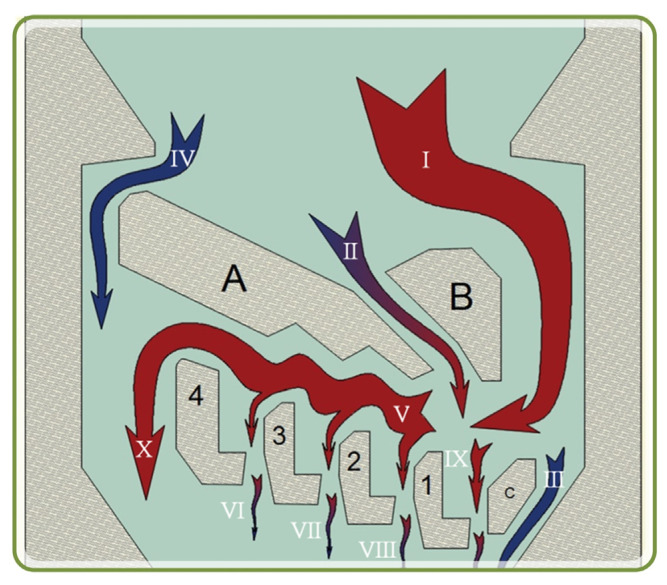
The trapping chamber flows schematics of the final design: (**A**–**C**)—flow breaking structures to form main streams; 1, 2, 3, & 4—L-shaped elements for capturing a single cell and holding it during the analyses of single RBC responses to a solution/object/stimuli; I–IX—flow streams. IV—the top by-passing collateral flow, II—the local turbulence flow that creates inertia moment pushing the cells towards the traps; III and IV—valve-like streams which dump excessive pressure; I—main inflow (red arrow); VI, VII, VIII, IX—weak flows to insure single cell will flow into the trap and stay there; X—main flow that passes traps and meets collateral flow IV and creates the main flow that goes into the next chamber.

The trapping geometry was designed in Autocad (CAD) and flow patterns were simulated in COMSOL Multiphysics. They are redesigned and re-simulated until all key flow parameters and constraints were satisfied. Two criteria were set: velocities in the slits to be close to 0.015 m/s, and a TVR value of 1.93 (chosen after analyzing the first 100 of the simulations as the minimum values). The final trapping chamber design is shown in Figure 4, where A, B, and C were the flow breaking structures to form main chamber streams; bodies 1, 2, 3, and 4 were L-shaped elements for capturing a single cell and holding it during the analyses of single RBC responses to a solution/object/stimuli; I-IX are the flow streams. IV is the top by-passing collateral flow. II is the local turbulence flow that creates the inertia moment pushing the cells towards the traps. III and IV are the valve-like streams that dump excessive pressure and I is the main inflow (large red arrow). VI, VII, VIII, and IX are weak flows to ensure single cell will flow into the trap and stay there. X is the main flow that passes the traps and meets collateral flow IV and creates the main flow that goes into the next chamber.

To ensure the correctness of the obtained solutions, the geometry-based and flow-based constraints were involved in the optimization. The geometry-based constraints include self-intersection, minimal inter-polygon distance, and other simple checks. The flow-based constraints validate the different parameters of liquid solution in COMSOL. The value of CRL was calculated directly and with an upper limit CRL < 30,000 (chosen after analyzing the first 100 of the simulations as the maximum value). Beyond this value, the flow had multiple turns with sharp angles causing cells to pile up around the pivotal points to block the passage. The value of CVR was calculated directly and with an upper limit CVR < 7 × 10^7^. The flow constraint 1.22 < TVR < 2 was calculated from the COMSOL liquid velocity magnitude field U (Equation (3)). The values for the constraints were chosen after analyzing the first 100 of the simulations as the minimum/maximum values to achieve the optimal slit through the flow vales of 0.015 m/s (COMSOL integral probe); designs that violate the constraints were rejected during the application of evolutionary operators.

The additional improvement of the computational performance is achieved using the parallelization of implemented operators. The probability of mutation and crossover was selected as 0.6 and 0.4. The size of the population was set as 300 and the maximum number of generations was defined as 100. Hyper parameters of evolutionary algorithm are chosen based on the best practices in the field of the geometrical structures design [28]. The through-slit velocities and parameters for the resulting geometries are listed in Table 1. The initial values of the “empty” (no obstacles) design in Figure 1e of vi, CRL, CVR, and TVR are obtained during simulations. Lines connecting the vertices at the end of the slits served as boundary edges.

The optimized values of Table 1 were obtained in the same way at the same locations but after the optimized obstacles were generated by the evolutionary algorithm and placed before the cell traps. Gain (%) is the percentage increase of vi, CRL, and TVR values compared with optimal values, where corresponding initial values were set as 100%.

### 3.2. Experimental Results

**Figure 5:**
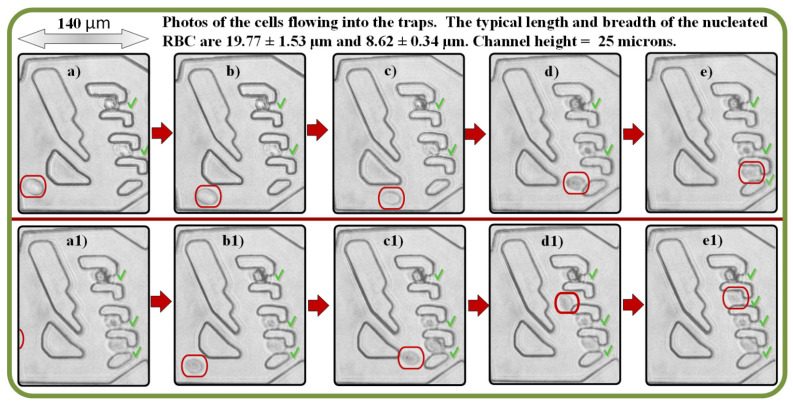
Experimental results of the prototype system following the evolutionary algorithm design to trap RBCs. The RBC cell approaching a trap is highlighted with the red oval. (**a**–**e**) Trapping of three living cells with one empty slot; (**a**–**d**) and (**a1**–**d1**) A single RBC is approaching the trap; (**e1**) the trapping is completed with all 4 slots occupied by erythrocytes. Green “V”s mark the successfully trapped cells in each slot. Time elapsed images from (**a**) to (**e**) take 11 s and it takes 3 s from (**a1**) to (**e1**); photos made with objective ×20 with NA 0.45, camera Opax A3514DU3, and Motic MHG-100B microscope.

Figure 5 shows the fabricated device designed by the evolutionary algorithm successfully trapping nucleated RBCs within one FOV (objective ×20 with NA 0.45) made with camera Opax A3514DU3 (Opax; Berkeley, CA, USA) and Motic MHG-100B microscope (Motic, Berkeley, CA, USA). Figure 5a–e show the trapping sequence of three living cells. Figure 5a–c,e,a1–d1 show a single RBC approaching and being trapped. Figure 5e1 has the trapping results, where all 4 slots were occupied by a single erythrocyte. Green “V”s mark the successfully trapped cell in each slot. Time elapsed images from Figure 5a to Figure 5e take 11 s and Figure 5a1 to Figure 5e1 take 5 s.

**Figure 6:**
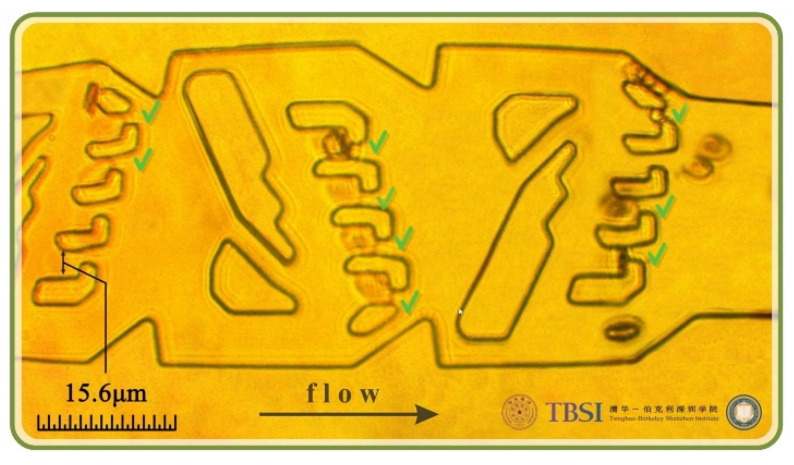
Experimental results of the prototype system following the evolutionary algorithm design to trap RBCs. Three consecutive chambers are designed to trap RBCs. Green “V”s mark the successfully trapped cells in each slot.

Figure 6 shows the experimental results of the prototype system following the evolutionary algorithm design to trap RBCs with three consecutive flow chambers. Green “V”s mark the successfully trapped cells.

The experiment was conducted under the following parameters: the width of the chamber inlet of 118 μm for 4 traps, and the height of the channels of 25 μm (less than the 2 × RBC width,) with the height/width ratio of 0.211 (which is within the recommend domain of PDMS manufacturer’s guidelines). Flow rates from 0.0046 to 621.4 μL/min were applied to the chamber inlet (max and min of the pump output). Applying this flow rate range is equivalent to applying a flowrate range of 0.046 μL/min to 6214 μL/min to a chip comprised of 10 branch channels. During the experiments, the viability of the cells was observed to be high enough for the RBCs to squeeze through the slits half their size and return to their original biconcave shape without turning into any damaged morphology under the high pressure.

The chamber inflow velocities are from 0.026 μm/s to 3510.6 μm/s (max and min of the pump output). The evolutionary algorithm parameters achieved for the best optimized solution are: CRL = 20,615 (62% increase compared to initial zero trapped cells design) in Figure 2e; CVR = 1.7 × 10^7^ (initial value 7.07 × 10^7^); TVR = 1.93 (initial, zero trapped cells design = 1.22, goal value = 1.93 fully achieved).

The evolutionary algorithm results applied to microfluidic trap design can be used for various design goals as well. For example, these results can help researchers to achieve the required flowrate parameters through the slits/channel designs. The evolutionary algorithm reduced the time required for microfluidics design and allowed for achieving desired flows with tight constraints. The setup time for the evolutionary algorithm was 40 human-hours and the computational cost for a 6-core Intel CPU was 60 h (100 generations). Furthermore, from a bio-physical viewpoint, the proposed method enabled the investigation and control of the transfer processes by RBCs in a dynamic mode.

## 4. Conclusions

In this paper, we presented a hydrodynamic trap with a unique feature to trap single RBCs in the flow channels by allowing through-slit flows. The single RBC trapping slits were designed with an evolutionary algorithm for the topology optimizations of the system. After adequate training, our system achieved desired flow parameters and met all constraints. Experimentally, we have built microfluidic devices and tested their trapping capabilities using frog RBCs for validations. Experimental results showed 4 out of 4 nucleated RBCs were trapped within one FOV.

We envision this evolutionary algorithm method can be applied to other microfluidics designs. In the future, the convergence speed of the algorithm can be further improved with expert-generated initial assumptions. In addition, the deep learning model can be applied to build the hybrid algorithms that can be used to achieve the better effectiveness of the whole system [29]. The designed cell-trapping system can be used in the microscopic studies of single cells in blood plasma flows, such as erythrocytes and lymphocytes. The automated design approach made it possible to fine-tune existing configurations and produce entirely new setups for each specific task.

## 5. Code and Data Availability

The scripts and data for the described implementation of generative design for single red blood cell traps were available in the open repository https://github.com/ITMO-NSS-team/rbc-traps-generative-design (accessed on 23 December 2021). The algorithmic implementation of evolutionary optimization of geometrically-encoded structures were added to the self-developed GEFEST framework: https://github.com/ITMO-NSS-team/GEFEST (accessed on 23 December 2021).

## Figures and Tables

**Table 1:** Values of velocities, optimization objectives, and obtained gains for the initial, and optimized solutions.

Parameter	Units	Initial	Target Values	Optimized	Gain, %
vl_1	m/s	0.012038	determined	0.02308	**92**
vl_2	m/s	0.009443		0.01579	**67**
vl_3	m/s	0.009478	by	0.012701	**34**
vl_4	m/s	0.009544		0.010092	**6**
vl_PD	m/s	0.005998	TVR ratio	0.012438	**107**
vl_main	m/s	0.027247		0.019577	**−28**
CVR	1/m	70,769,000	<7 × 10^7^	17,113,000	**−76**
CRL	1/s	12,717	<30,000	20,615	**62**
TVR (target)	**-**	**1.22**	**1.22 < TVR < 2**	**1.93**	**58**

## Data Availability

Not applicable.
